# Genetic variants in nuclear DNA along with environmental factors modify mitochondrial DNA copy number: a population-based exome-wide association study

**DOI:** 10.1186/s12864-018-5142-7

**Published:** 2018-10-16

**Authors:** Zhihua Li, Meng Zhu, Jiangbo Du, Hongxia Ma, Guangfu Jin, Juncheng Dai

**Affiliations:** 1 Department of Epidemiology, Center for Global Health, School of Public Health, Nanjing Medical University, Nanjing, 211166 Jiangsu China; 2 Jiangsu Key Lab of Cancer Biomarkers, Prevention and Treatment, Collaborative Innovation Center for Cancer Personalized Medicine, Nanjing Medical University, Nanjing, 211166 Jiangsu China; 3 Department of Thoracic Surgery, The First Affiliated Hospital of Nanjing Medical University, Nanjing, 210029 Jiangsu China

**Keywords:** MtDNA copy number, Genetic variants, Exome-wide association study, PM_2.5_ exposure

## Abstract

**Background:**

Mitochondrial DNA (mtDNA) copy number has been found associated with multiple diseases, including cancers, diabetes and so on. Both environmental and genetic factors could affect the copy number of mtDNA. However, limited study was available about the relationship between genetic variants and mtDNA copy number. What’s more, most of previous studies considered only environmental or genetic factors. Therefore, it’s necessary to explore the genetic effects on mtDNA copy number with the consideration of PM_2.5_ exposure and smoking.

**Results:**

A multi-center population-based study was performed with 301 subjects from Zhuhai, Wuhan and Tianjin. Personal 24-h PM_2.5_ exposure levels, smoking and mtDNA copy number were evaluated. The Illumina Human Exome BeadChip, which contained 241,305 single nucleotide variants, was used for genotyping. The association analysis was conducted in each city and meta-analysis was adopted to combine the overall effect among three cities. Seven SNPs showed significant association with mtDNA copy number with *P* value less than 1.00E-04 after meta-analysis. The following joint analysis of our identified SNPs showed a significant allele-dosage association between the number of variants and mtDNA copy number (*P* = 5.02 × 10^− 17^). Further, 11 genes were identified associated with mtDNA copy number using gene-based analysis with a *P* value less than 0.01.

**Conclusion:**

This study was the first attempt to evaluate the genetic effects on mtDNA copy number with the consideration of personal PM_2.5_ exposure level. Our findings could provide more evidences that genetic variants played important roles in modulating the copy number of mtDNA.

**Electronic supplementary material:**

The online version of this article (10.1186/s12864-018-5142-7) contains supplementary material, which is available to authorized users.

## Background

Mitochondria are vital eukaryotic organelles and participate in various physiological processes, including energy supplying, oxidative phosphorylation, cell apoptosis and so on [1, 2]. Mitochondria have genetic materials independent of the host nuclear genome, known as mitochondrial DNA (mtDNA). Usually, each mitochondrion carries 2–10 copies of mtDNA in human cells [3]. However, the mtDNA are easily damaged because of the absence of DNA repair machinery. As a result, mitochondrion alter its copy number as a compensation for mtDNA damage [4]. Many studies have proved that variation of mtDNA copy number could modify the susceptibility of cancers, diabetes and infertility [5–7]. Nowadays, both genetic and exogenous factors have been found associated with the copy number of mtDNA [8–10].

Among the exogenous factors, exposure of PM_2.5_ (fine particulate matter) is ubiquitous and inevitable for peoples. The situation is especially serious in china due to the rapid economic development recent years [11]. PM_2.5_ is also known as fine particulate matter and is a mixture of organic chemicals and transition metals. Previous studies suggested that high PM_2.5_ exposure was associated with decreased mtDNA copy number [12, 13]. In addition to exogenous factors, genetic factors could also modulate the copy number of mtDNA. Curran and Xing et al. found that mtDNA content appeared to have a high heritability [14, 15]. Furthermore, some candidate genes (such as *TFAM*, *TIMM2*3 and *PARL*) and single nucleotide polymorphisms (SNPs) could also influence the mtDNA copy number [14, 16]. In 2014, Lopez and colleagues performed the first genome-wide association study (GWAS) of mtDNA copy number and identified 15 significant SNPs using 386 subjects from Spanish [17]. However, the environmental factors (such as PM_2.5_ exposure level and smoking) were not considered in previous study and these genetic variants only explained a small fraction of the total variation. What’s more, the genetic background might be different between Spanish and Chinese population. Therefore, more efforts are warranted to evaluate the association between genetic variants and mtDNA copy number in Chinese population.

In the present study, we performed a multi-center population-based study with 301 subjects from three cities to evaluate the association between genetic variants and mtDNA copy number. Effects of PM_2.5_ exposure level and smoking pack-years on mtDNA copy number were also assessed. The quantitative real-time PCR was used to measure the mtDNA copy number of peripheral blood leucocyte and 241,305 SNPs were genotyped using the Illumina Human Exome Beadchip.

## Methods

### Study subject

Subjects in this study were exactly the same with that in our previous studies [18, 19]. In brief, 307 subjects with different PM_2.5_ exposure level from Zhuhai, Wuhan and Tianjin were included in this study. After signing the informed consent, each participant provided 5-mL peripheral blood for genotyping and measurement of mtDNA copy number. The demographic information and smoking information were collected using a unified questionnaire. The Ethics and Human Subject Committees of Tongji Medical College and Nanjing Medical University approved this study. The basic information of the subjects was summarized in Table 1.

**Table 1:** Demographic and basic information of subjects in Zhuhai, Wuhan and Tianjin

Characteristics	Zhuhai (*N* = 108)	Wuhan (*N* = 114)	Tianjin (*N* = 79)
Categorical variables			
Gender			
Male	36 (33.33%)	53 (46.49%)	32 (40.51%)
Female	72 (66.67%)	61 (53.51%)	47 (59.49%)
Smoking status			
Ever	18 (16.67%)	47 (41.23%)	25 (31.65%)
Never	90 (83.33%)	67 (58.77%)	54 (68.35%)
Continuous variables			
Age (years)	53.11 ± 6.76	51.37 ± 6.19	66.61 ± 5.53
Cumulative smoking(pack-years)^a^	24 (14.48–41.50)	16 (9.50–30.50)	29 (18–42)
PM_2.5_(μg/m^3^)^a^	68.36 (37.17–115.94)	114.96 (86.68–153.25)	146.60 (88.63–261.41)
mtDNA copy number^b^	−0.431(− 1.083, 0.491)	0.251(− 0.372, 0.925)	− 0.049 (− 0.500, 0.368)

^a^The median (25–75%) of pack-years and PM_2.5_ exposure levels in each city;

^b^The median (25–75%) of mtDNA copy number after taking INT transformation;

### Monitoring for PM_2.5_ exposure level

The monitoring for personal 24-h real-time PM_2.5_ exposure has been described previously [18, 19]. Briefly, the Sensidyne Company sampler pump and 37-mm Teflon filters from Beijing Lianyi Xingtong Apparatus & Instrument Co., Ltd. were used to measure the PM_2.5_ exposure level. The flow rate was set at 2.0 L/min for 24 h. The filter was weighted before and after sampling. We calculated the PM_2.5_ concentration based on the equation shown below, where PM_2.5_ concentration (ug/m^3^) was represented by C, m_1_ and m_2_ represented the weight of filter (mg) before and after sampling, V was the flow rate (2 L/min in this study) and t was the sampling time (24 h × 60 min/h = 1440 min in this study).

$$ C=\frac{m2-m1}{V\times t} $$

### Measurement of mitochondria DNA copy number

We used the phenol/chloroform to extract the genomic DNA from peripheral blood leucocyte. Further, we measured the relative mtDNA using qPCR (7900HT Real Time PCR system, Applied BiosystemsTM, Lincoln Centre Drive Foster City, CA). In brief, we designed two primer pairs, one for mitochondrial subunit ND1 gene (*MT-ND1*, primer sequences: F, 5′-CCCTAAAACCCGCCACATCT-3′ and R, 5′-GAGCGATGGTGAGAGCTAAGGT-3′) and another for nuclear gene human globulin (*HGB*, primer sequences: F, 5′-GAAGAGCCAAGGACAGGTAC-3′ and R, 5′-CAACTTCATCCACGTTCACC-3′) [20]. A 10 ul reaction biosystem was constructed with a final DNA concentration of 5 ng/ul. The thermal cycling procedure was set as follows: 50 °C for 2 min, then 95 °C keeping 2 min, followed by 40 cycles of 95 °C for 15 s and 60 °C for 1 min (*MT-ND1*) or 56 °C for 1 min (*HGB*). Relative mtDNA was calculated using the ratio of *MT-ND1* to *HGB* based on the standard curves. All the samples were measured in triplicates and the average value was reported. For each sample, the ratio of *MT-ND1* to *HGB* was calculated through subtracting the *HGB* Ct value from *MT-ND1* Ct value (-dCt). Furthermore, the relative ratio of *MT-ND1* to *HGB* (-ddCt) could be calculated by subtracting the –dCt of the calibrator DNA from the ratio of each sample. Finally, we calculated the relative mtDNA copy number using the formula: 2 × 2^−ddCt^ [5].

### Genotyping and quality control (QC)

In this study, the genotyping was performed using Illumina Human Exome BeadChip, which contained 241,305 SNVs (single nucleotide variants) around exonic regions. Systematic quality control was performed before the association analysis. As far as it concerns samples, six samples (two samples from Zhuhai and four samples from Wuhan) with call rates less than 95% were excluded; SNVs that satisfied any of the following criteria would be removed: (1) non-autosomal; (2) genotyping call rate < 95%; (3) Hardy-Weinberg equilibrium (HWE) < 0.001. As a result, 301 qualified subjects with 238,927 SNVs were kept for further analysis.

### Statistical analysis

The PM_2.5_ exposure level and relative mtDNA copy number were described using the 25%, 50% and 75% percentiles. The HWE test was performed using goodness-of-fit χ^2^ test. Considering the abnormal distribution of mtDNA copy number, it was transformed using the rank-based inverse-normal transformation (INT) [21]. The multivariable linear regression model was used to evaluate the association between genetic variants and mtDNA copy number. The additive genetic model was adopted. Age, gender, PM_2.5_ exposure level and pack-years of smoking were adjusted to control their potential confounding. The association analysis was performed individually in each city and combined result of these three cities was calculated using meta-analysis. SNPs with consistent association direction in three cities and *P* value less than 1 × 10^− 4^ were recognized as significant variants [22, 23]. Further, we used the multivariate stepwise regression model to screen the independent factors of mtDNA copy number using Stata 11. Variables with *P* < 0.05 would be reserved in this model.

Functional annotations were performed based on four public databases, including RegulomeDB (http://regulome.stanford.edu/), HaploReg v4.1(https://pubs.broadinstitute.org/mammals/haploreg/haploreg.php), GTEx V7 (https://www.gtexportal.org/home/) and CADD (http://cadd.gs.washington.edu/home). The gene-based analysis was conducted using SKAT-O method (SNV-set (sequence) kernel association test) [24]. The association analysis was performed using plink 1.9 and R 3.3.3.

## Results

### Characteristics of study subjects

Characteristics of 301 subjects (108 subjects from Zhuhai, 114 subjects from Wuhan and 79 subjects from Tianjin) were summarized in Table 1. The mean age of subjects in Tianjin was 66.61 ± 5.53 years old, larger than that in Zhuhai (53.11 ± 6.76 y) and Wuhan (51.37 ± 6.19 y). Meanwhile, the PM_2.5_ exposure level in Tianjin was also the highest among these three cities (median for Tianjin: 146.60 μg/m^3^; median for Zhuhai: 68.36 μg/m^3^; median for Wuhan: 114.96 μg/m^3^). Subjects in Tianjin had higher smoking pack-years (29 pack-years) than that in Zhuhai (24 pack-years) and Wuhan (16 pack-years). The median mtDNA copy number of study subjects after taking INT was − 0.431 (− 1.083, 0.491), 0.251 (− 0.372, 0.925) and − 0.049 (− 0.500, 0.368) for Zhuhai, Wuhan and Tianjin, respectively.

### Association between smoking, PM_2.5_ exposure level and mtDNA copy number

In this study, we evaluated the effect of smoking and PM_2.5_ exposure on mtDNA copy number. As shown in Additional file 1: Figure S1, the median mtDNA copy number in smokers was significantly higher than that in non-smokers (*P* = 0.025), suggesting that smoking could be associated with increased mtDNA copy number. This result was further proved through the linear regression model with adjustment for age, gender and PM_2.5_ exposure level (β = 0.448, *P* = 0.012, Additional file 2: Table S1). Further, we divided these subjects into high PM_2.5_ exposure subgroup and low PM_2.5_ exposure subgroup in each city based on the median PM_2.5_ exposure level. In Zhuhai, the median mtDNA copy number in high PM_2.5_ exposure subgroup was lower than that in low PM_2.5_ exposure subgroup (*P* = 0.001). Result from the regression model showed that PM_2.5_ was significantly associated with decreased copy number of mtDNA in Zhuhai (*P* = 0.024, Additional file 2: Table S1). However, we did not observe the consistent results in Wuhan and Tianjin. Meta-analysis indicated that PM_2.5_ was negatively correlated with mtDNA copy number, but the association was not statistically significant (*P* = 0.241).

### Association between genetic variants and mtDNA copy number

**Fig. 1:**
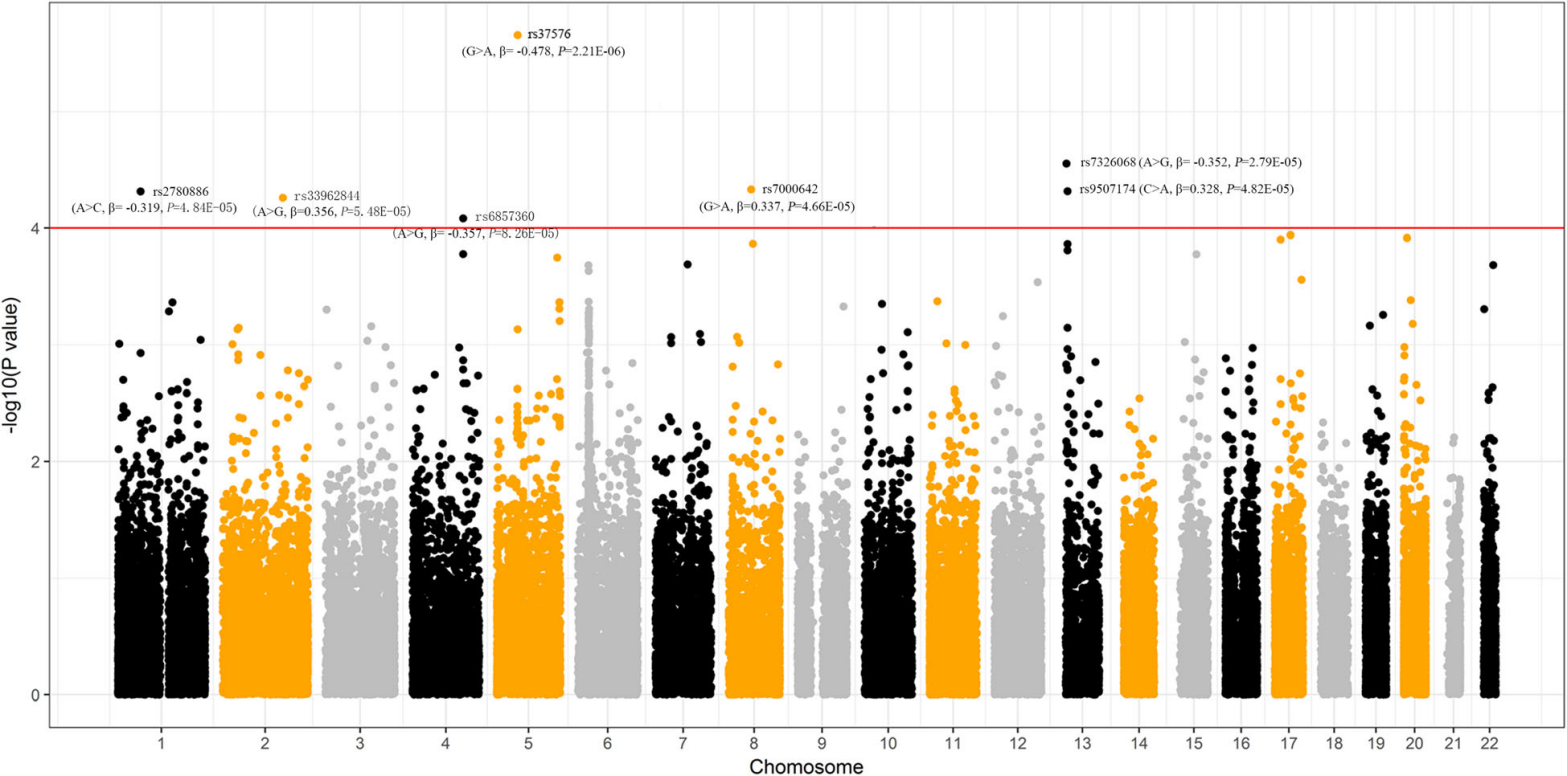
The manhattan plot of association between genetic variants and mtDNA copy number

Totally, 238,927 SNVs were kept for the association analysis using linear regression model with the adjustment for age, gender, pack-years and PM_2.5_ exposure level (Fig. 1). Among them, 13,027 SNVs showed consistent direction of regression coefficients in these three cities. Further, 7 SNPs showed significant association with mtDNA copy number with *P* value less than 1.00E-04 after taking meta-analysis (Table 2, Fig. 1). SNP rs37576 (located in *PDE4D*, 5q11.2, G > A, β = − 0.478, *P* = 2.21E-06) showed the most significant association. Furthermore, we used the multivariable stepwise regression analysis to identify the independent factors that could modulate the mtDNA copy number. Our identified 7 significant SNPs along with age, gender, pack-years and PM_2.5_ exposure level were analyzed. Finally, only the 7 SNPs were reserved in the stepwise regression model (Table 3), suggesting that these 7 SNPs could influence the copy number of mtDNA independently.

**Table 2:** Association between 7 significant SNPs and mtDNA copy number in three cities and meta-analysis

CHR	SNP	BP	Gene	Effect	Zhuhai		Wuhan		Tianjin		Meta		
					β^a^	*P* ^a^	β^a^	*P* ^a^	β^a^	*P* ^a^	β^b^	*P* ^b^	*P*_*het*_^c^
5q11.2	rs37576	58830145	*PDE4D*	A	−0.519	7.80 × 10^−3^	− 0.518	2.78 × 10^− 3^	−0.407	1.74 × 10^−2^	− 0.478	2.21 × 10^−6^	0.963
13q12.11	rs7326068	21209512	*IFT88*	G	−0.291	5.32 × 10^−2^	−0.275	5.57 × 10^−2^	− 0.494	1.18 × 10^−3^	−0.352	2.79 × 10^−5^	0.703
8q12.3	rs7000642	64428514	*IFITM8P*	A	0.360	1.51 × 10^−2^	0.348	1.77 × 10^−2^	0.305	3.27 × 10^−2^	0.337	4.66 × 10^−5^	0.994
13q12.12	rs9507174	24429097	*MIPEP*	A	0.360	1.48 × 10^−2^	0.218	7.62 × 10^−2^	0.485	3.76 × 10^−3^	0.328	4.82 × 10^−5^	0.614
1p31.3	rs2780886	65331897	*JAK1*	C	−0.407	3.81 × 10^−3^	−0.393	4.43 × 10^−3^	−0.159	0.246	−0.319	4.84 × 10^−5^	0.552
2q31.1	rs33962844	171260797	*MYO3B*	G	0.539	1.52 × 10^−3^	0.385	9.56 × 10^−3^	0.176	0.241	0.356	5.48 × 10^−5^	0.436
4q31.21	rs6857360	144445789	*SMARCA5*	G	−0.476	4.03 × 10^−3^	− 0.370	1.34 × 10^−2^	−0.218	0.189	−0.357	8.26 × 10^−5^	0.740

^a^Association was evaluated using additive genetic model with adjustment for age, gender, smoking pack-years and PM_2.5_ exposure level;

^b^Results of meta-analysis based on fixed-effect model;

^c^*P* value for heterogeneity test;

**Table 3:** The independent factors that could modulate mtDNA copy numbers using multivariate stepwise regression analysis

Variables	Effect	β^a^	95% CI		SE	*P*
			Lower	Upper		
rs7000642	A	0.212	0.061	0.364	0.077	0.006
rs7326068	G	−0.218	− 0.374	−0.062	0.079	0.006
rs37576	A	−0.250	−0.445	− 0.056	0.099	0.012
rs9507174	A	0.218	0.070	0.366	0.075	0.004
rs2780886	C	−0.239	−0.384	−0.095	0.074	0.001
rs33962844	G	0.285	0.119	0.451	0.084	0.001
rs6857360	G	−0.219	−0.386	−0.052	0.085	0.010

^a^Multivariate stepwise regression analysis was performed using Stata 11, variables with *P* value less than 0.05 were reserved in this model, otherwise would be excluded

**Fig. 2:**
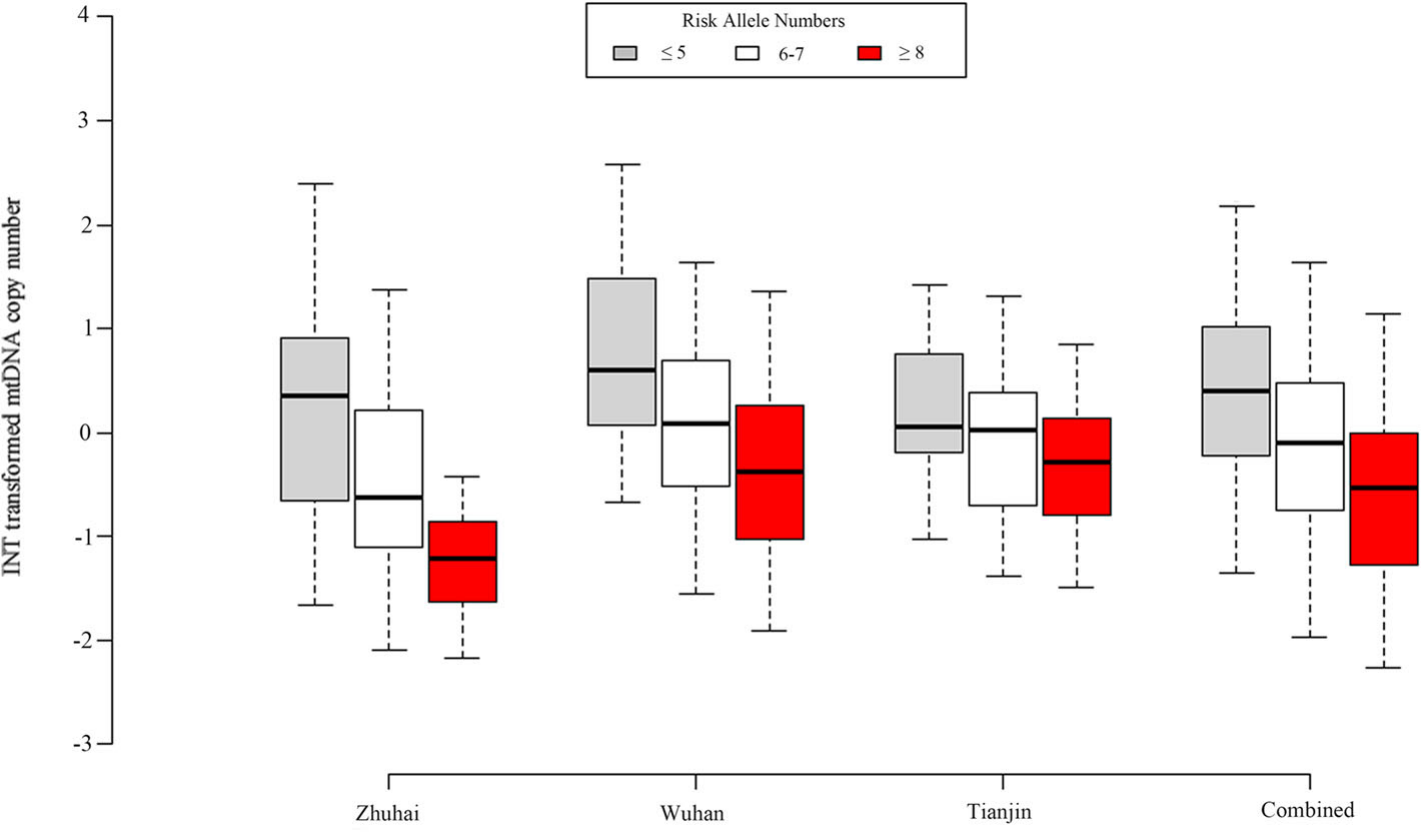
Joint effect of the 7 identified SNPs on mtDNA copy number in each city and combined analysis

In the interest of exploring the cumulative effect of our identified 7 SNPs on mtDNA copy number, we performed a joint analysis. All the subjects were divided into three subgroups: “≤5”, “6–7” and “≥8” according to their carried effect allele numbers in each city. We observed a significant allelic dosage-effect of combined 7 SNPs on mtDNA copy number in all the three cities (*P* for trend was 6.99E-09, 1.78E-06 and 2.40E-03 for Zhuhai, Wuhan and Tianjin, respectively). When we combined the three cities, the similar significant dosage-effect tendency was observed (*P* for trend: 5.02E-17, Table 4 and Fig. 2).

**Table 4:** The cumulative effects of our identified 7 significant SNPs on mtDNA copy number

Number^a^	Zhuhai				Wuhan				Tianjin				Combined			
	N^d^	MCN^b^	β^c^	*P* ^c^	N^d^	MCN^b^	β^c^	*P* ^c^	N^d^	MCN^b^	β^c^	*P* ^c^	N^d^	MCN^b^	β^c^	*P* ^c^
≤5	51	0.350 (−0.641, 0.885)	Reference		52	0.596 (0.106, 1.475)	Reference		30	0.050 (−0.189, 0.744)	Reference		133	0.390 (−0.221, 1.011)	Reference	
6–7	39	−0.572 (−1.076, 0.219)	− 0.715	2.070E-04	39	0.091 (−0.519, 0.688)	− 0.663	8.96E-04	22	0.025 (−0.663, 0.375)	− 0.332	0.173	100	−0.099 (− 0.746, 0.467)	−0.606 (− 0.835, − 0.376)	2.33E-07
≥8	17	− 1.216 (− 1.605, − 0.902)	−1.478	4.04E-08	23	−0.381 (−1.029, 0.251)	− 1.087	5.93E-06	26	− 0.281 (− 0.751, 0.085)	−0.715	2.55E-03	66	−0.528 (− 1.278, − 0.008)	−1.070 (− 1.340, − 0.808)	2.08E-15
*P* for trend	107		− 0.734	6.99E-09	114		− 0.560	1.78E-06	78		−0.357	2.40E-03	299		−0.548 (− 0.676, − 0.420)	5.02E-17

^a^Subgroups were divided according to the carried risk allele numbers of each subject

^b^The median (25–75%) of the mtDNA copy number after taking INT transformation in each subgroup

^c^This analysis was performed with the adjustment for age, gender, PM_2.5_ exposure and smoking pack-years in each subgroup or combined samples

^d^Sample size in each subgroup

### Gene-based analysis

In this study, we further analyzed the overall effects of SNPs that located in the same genes using SKAT package. In total, 995 genes with at least three variants were evaluated. Among them, 11 genes showed significant association with mtDNA copy number (*P* value < 0.01, Table 5). The *XIRP1* showed the most significant association with mtDNA copy number with a *P* value of 6.54E-04.

**Table 5:** Eleven candidate genes that showed significant association with mtDNA copy number based on gene-based analysis (*P* value < 0.01)

Gene	Chr	*P*	Q^a^	N_All^b^	N_Test^c^	N_Rare^d^	N_Common^e^
*XIRP1*	3	6.54E-04	7.4	3	3	1	2
*RASGRP3*	2	1.18E-03	1202.5	9	9	0	9
*RFX2*	19	3.46E-03	5.8	5	5	2	3
*CTAGE5*	14	3.49E-03	31725.3	3	3	3	0
*CENPF*	1	3.55E-03	5.7	18	18	1	17
*CREBBP*	16	5.02E-03	450.2	3	3	0	3
*TBC1D8*	2	7.25E-03	487.7	3	3	0	3
*ACSBG2*	19	7.25E-03	5.0	3	3	2	1
*GBP4*	1	8.72E-03	735.1	4	4	0	4
*NPLOC4*	17	9.30E-03	747.9	4	4	0	4
*TNR*	1	9.64E-03	469.8	3	3	0	3

^a^Q value was the test statistic;

^b^Total number of SNPs in each gene;

^c^Number of SNPs that were contained in the test;

^d^Number of SNPs with rare frequencies;

^e^Number of common SNPs

### Functional annotation

For our identified 7 significant SNPs, we performed functional annotations using multiple databases. Functional annotations indicated that rs7326068 and rs33962844 were located in transcript factors binding sites or DNase peak according to RegulomeDB website, suggesting that these two SNPs might modify the binding of transcript factors (Additional file 2: Table S2). Most of these 7 SNPs could change the motifs and regulate the expression of surrounding genes. Notably, SNP rs33962844 (*MYO3B*, A > G) was a missense with a CADD score of 13.98, indicating that this variant could be harmful to human genome. Consistently, this missense was predicted to be deleterious with a score of − 4.90 based on PROVEAN database.

## Discussion

In the current study, we performed an exome-wide association study to assess the association between genetic variants and mtDNA copy number with the consideration of personal PM_2.5_ exposure levels. Seven SNPs and 11 genes were identified significantly associated with mtDNA copy number. Our findings provided more evidences that genetic variants in nuclear DNA could modulate the copy number of mtDNA.

Up till now, more and more studies proved that variation of mtDNA copy number was closely related to various diseases [5, 25, 26]. Therefore, studies on the influence factors of mtDNA copy number are vital for the prevention and treatment of mtDNA related diseases. In previous studies, high PM_2.5_ exposure was found to be associated with decreased mtDNA copy number [12, 13]. This was consistent with our finding in Zhuhai, but not validated in Wuhan and Tianjin. Many factors could be responsible for this, such as the different components of PM_2.5_, differential concentration of PM_2.5_ and the other potential compounds [12]. Besides the PM_2.5_ exposure level, we also observed that smoking contributed to the increased mtDNA copy number, which was consistent with the report by Xing et al. [15]. Nowadays, studies about the roles of PM_2.5_ and smoking on mtDNA copy number are still limited, our findings could provide more clues for the following studies.

For our identified 7 SNPs, functional annotations indicated that most of them could regulate the expression of surrounding genes. SNP rs9507174 was located in 13q12.12 loci and showed significant association with the expression of *MIPEP* (mitochondrial intermediate peptidase) according to GTEx database. The *MIPEP* encoded proteins that participated in the maturation of oxidative phosphorylation (OXPHOS)-related proteins. These OXPHOS-related proteins targeted to the mitochondrial matrix or inner membrane, suggesting that *MIPEP* could influence the replication and expression of mtDNA [27–29]. In addition, *MIPEP* has been found associated with the risk of lung cancer and myopia [30, 31]. The underlying pathogenic mechanisms were not clear until now. Notably, several studies revealed that the increased mtDNA copy number contributed to the lung cancer risk [32, 33]. These findings suggested that the changed mtDNA copy number by *MIPEP* might be the potential disease-causing pathway (Additional file 3: Figure S2). Indeed, we observed a significant association between the rs9507174 and lung cancer risk using our previous data (C > A, OR = 1.10 (1.01–1.20), *P* = 0.037) [30].

SNP rs33962844 was located in 2q31.1, the 20th exon of *MYO3B* and was a missense mutation. According to PROVEAN database, SNP rs33962844 was predicted to be deleterious with a score of − 4.90. Consistently, the CADD score was 13.98, more than 96% of the human genetic variants, indicating that this genetic variant might be seriously harmful to human beings. The *MYO3B* encoded one of the ATPases and played important roles in the production and utility of ATP [34]. Similarly, SNP rs6857360 was associated with the expression of *SMARCA5*, who encoded a member of the SWI/SNF family and also had ATPase activities [35]. These evidences suggested that both *MYO3B* and *SMARCA5* could affect the oxidative phosphorylation and the normal supply of ATP. This might aggravate the burden of mitochondria and result in a series of change [36]. In addition, previous studies also indicated that *MYO3B* was associated with obesity and Kawasaki disease [37, 38]. As for *SMARCA5*, it was an important chromatin remodeling gene and has been found associated with breast cancer, Alzheimer’s disease and leukemia [39–41]. Interestingly, in our previous study, the rs6857360 (*SMARCA5*) was found associated with the DNA damage levels [42]. This finding suggested us that *SMARCA5* could influence both the nuclear and the mitochondrial DNA.

To date, there have been three articles focusing on the genome-wide association study of mtNDA copy number [17, 43, 44]. We attempted to compare our results with previous findings, however, no consistent finding was found. Many reasons could explain this point, such as different experimental measure of mtDNA, different genotype chips and populations, etc. In addition to 7 significant SNPs, we also identified 11 candidate genes that could modulate the mtDNA copy number based on gene-based analysis. Among these genes, *RASGRP3* encoded a guanine nucleotide exchange factor and played important roles in lupus and cancers [45, 46]. Besides, the *CREBBP* encoded the cAMP-response element binding protein and was involved in the transcriptional co-activation of many different transcription factors [47]. Numerous studies have proved that *CREBBP* could influence the susceptibility of cancers, Rubinstein-Taybi syndrome and diabetes [48–50]. As for the other genes, the related studies are limited and more studies are needed to reveal their functions.

Compared with previous studies, this study has two prominent advantages: (1) we systematically evaluated the association between genetic variants and mtDNA copy number with the consideration of personal PM_2.5_ exposure levels and smoking pack-years for the first time; (2) the study samples were recruited from the south, middle and north cities and results were validated in each city. However, this study also has some limitations. First, the relative sample size was limited because of the difficulty of carrying PM_2.5_ sampler keeping for 24 h. Second, the underlying mechanisms of our identified SNPs and genes were not well expounded. Studies with larger sample size and functional assays are warranted to validate our findings.

## Conclusions

This study was the first attempt to evaluate the genetic effects on mtDNA copy number with the consideration of smoking and personal PM_2.5_ exposure level. Seven significant SNPs and 11 genes were identified associated with the copy number of mtDNA. Our study provided more evidences that genetic variants in nuclear DNA along with environmental factors could modulate the mitochondrial DNA copy number.

## Additional files

Additional file 1: **Figure S1.** The effects of smoking and PM_2.5_ exposure level on mtDNA copy number. The first column indicated that smokers have higher mtDNA copy number than non-smokers. The 2–5 columns showed that the median mtDNA copy number in subjects with low and high PM_2.5_ exposure in each city and combined analysis. (DOCX 331 kb)

Additional file 2: **Table S1.** Association between PM_2.5_, smoking and mtDNA copy number. **Table S2.** Functional annotations for our identified 7 significant SNPs. (DOCX 21 kb)

Additional file 3: **Figure S2.** (a) Association between rs9507174 genotypes and mtDNA copy number; (b): Association between rs9507174 genotypes and the expression of *MIPEP* gene according GTEx database; (c): Association between rs9507174 genotypes and lung cancer risk using our previous data. The frequency of “A” allele of rs9507174 was higher in lung cancer patients; (d): Association between mtDNA copy number and lung cancer risk. This figure was made based on the result from Hosgood et al. ‘s paper. The horizontal axis was the quartile of mtDNA copy number and the vertical axis represents the Odds Ratio for lung cancer. (DOCX 496 kb)

## Abbreviations

GWAS: Genome-wide association study
INT: Inverse-normal transformation
mtDNA: Mitochondrial DNA
SNVs: Single nucleotide variants

## References

[1] Kroemer G, Galluzzi L, Brenner C (2007). Mitochondrial membrane permeabilization in cell death. Physiol Rev.

[2] Newmeyer DD, Ferguson-Miller S (2003). Mitochondria: releasing power for life and unleashing the machineries of death. Cell.

[3] Lee HC, Wei YH (2000). Mitochondrial role in life and death of the cell. J Biomed Sci.

[4] Shokolenko I, Venediktova N, Bochkareva A, Wilson GL, Alexeyev MF (2009). Oxidative stress induces degradation of mitochondrial DNA. Nucleic Acids Res.

[5] Hosnijeh FS, Lan Q, Rothman N, San Liu C, Cheng WL, Nieters A (2014). Mitochondrial DNA copy number and future risk of B-cell lymphoma in a nested case-control study in the prospective EPIC cohort. Blood.

[6] Lemnrau A, Brook MN, Fletcher O, Coulson P, Tomczyk K, Jones M (2015). Mitochondrial DNA copy number in peripheral blood cells and risk of developing breast Cancer. Cancer Res.

[7] Jiang M, Kauppila TES, Motori E, Li X, Atanassov I, Folz-Donahue K (2017). Increased Total mtDNA copy number cures male infertility despite unaltered mtDNA mutation load. Cell Metab.

[8] Shen M, Zhang L, Bonner MR, Liu CS, Li G, Vermeulen R (2008). Association between mitochondrial DNA copy number, blood cell counts, and occupational benzene exposure. Environ Mol Mutagen.

[9] Carugno M, Pesatori AC, Dioni L, Hoxha M, Bollati V, Albetti B (2012). Increased mitochondrial DNA copy number in occupations associated with low-dose benzene exposure. Environ Health Perspect.

[10] Lynch SM, Weinstein SJ, Virtamo J, Lan Q, Liu CS, Cheng WL (2011). Mitochondrial DNA copy number and pancreatic cancer in the alpha-tocopherol beta-carotene cancer prevention study. Cancer Prev Res.

[11] Guo H, Cheng T, Gu X, Wang Y, Chen H, Bao F (2017). Assessment of PM2.5 concentrations and exposure throughout China using ground observations. Sci Total Environ.

[12] Wong JYY, Hu W, Downward GS, Seow WJ, Bassig BA, Ji BT (2017). Personal exposure to fine particulate matter and benzo[a]pyrene from indoor air pollution and leukocyte mitochondrial DNA copy number in rural China. Carcinogenesis.

[13] Pieters N, Janssen BG, Dewitte H, Cox B, Cuypers A, Lefebvre W (2016). Biomolecular markers within the Core Axis of aging and particulate air pollution exposure in the elderly: a cross-sectional study. Environ Health Perspect.

[14] Curran JE, Johnson MP, Dyer TD, Goring HH, Kent JW, Charlesworth JC (2007). Genetic determinants of mitochondrial content. Hum Mol Genet.

[15] Xing J, Chen M, Wood CG, Lin J, Spitz MR, Ma J (2008). Mitochondrial DNA content: its genetic heritability and association with renal cell carcinoma. J Natl Cancer Inst.

[16] Curran JE, Jowett JB, Abraham LJ, Diepeveen LA, Elliott KS, Dyer TD (2010). Genetic variation in PARL influences mitochondrial content. Hum Genet.

[17] Lopez S, Buil A, Souto JC, Casademont J, Martinez-Perez A, Almasy L (2014). A genome-wide association study in the genetic analysis of idiopathic thrombophilia project suggests sex-specific regulation of mitochondrial DNA levels. Mitochondrion.

[18] Liu J, Xie K, Chen W, Zhu M, Shen W, Yuan J (2017). Genetic variants, PM2.5 exposure level and global DNA methylation level: A multi-center population-based study in Chinese. Toxicology Letters.

[19] Chu M, Sun C, Chen W, Jin G, Gong J, Zhu M (2015). Personal exposure to PM2.5, genetic variants and DNA damage: a multi-center population-based study in Chinese. Toxicol Lett.

[20] Zhu X, Mao Y, Huang T, Yan C, Yu F, Du J (2017). High mitochondrial DNA copy number was associated with an increased gastric cancer risk in a Chinese population. Mol Carcinog.

[21] Bishara AJ, Hittner JB (2012). Testing the significance of a correlation with nonnormal data: comparison of Pearson, spearman, transformation, and resampling approaches. Psychol Methods.

[22] Shukla S, Evans JR, Malik R, Feng FY, Dhanasekaran SM, Cao X (2017). Development of a RNA-Seq Based Prognostic Signature in Lung Adenocarcinoma. Journal of the National Cancer Institute.

[23] Smyth DJ, Plagnol V, Walker NM, Cooper JD, Downes K, Yang JH (2008). Shared and distinct genetic variants in type 1 diabetes and celiac disease. N Engl J Med.

[24] Ionita-Laza I, Lee S, Makarov V, Buxbaum JD, Lin X (2013). Sequence kernel association tests for the combined effect of rare and common variants. Am J Hum Genet.

[25] Zhang Y, Guallar E, Ashar FN, Longchamps RJ, Castellani CA, Lane J (2017). Association between mitochondrial DNA copy number and sudden cardiac death: findings from the atherosclerosis risk in communities study (ARIC). Eur Heart J.

[26] Otten AB, Smeets HJ (2015). Evolutionary defined role of the mitochondrial DNA in fertility, disease and ageing. Hum Reprod Update.

[27] Chew A, Buck EA, Peretz S, Sirugo G, Rinaldo P, Isaya G (1997). Cloning, expression, and chromosomal assignment of the human mitochondrial intermediate peptidase gene (MIPEP). Genomics.

[28] Branda SS, Isaya G (1995). Prediction and identification of new natural substrates of the yeast mitochondrial intermediate peptidase. J Biol Chem.

[29] Gustafsson CM, Falkenberg M, Larsson NG (2016). Maintenance and expression of mammalian mitochondrial DNA. Annu Rev Biochem.

[30] Hu Z, Wu C, Shi Y, Guo H, Zhao X, Yin Z (2011). A genome-wide association study identifies two new lung cancer susceptibility loci at 13q12.12 and 22q12.2 in Han Chinese. Nat Genet.

[31] Shi Y, Qu J, Zhang D, Zhao P, Zhang Q, Tam POS (2011). Genetic variants at 13q12.12 are associated with high myopia in the Han Chinese population. Am J Hum Genet.

[32] Hou YL, Chen JJ, Wu YF, Xue CJ, Li FZ, Zheng Q (2013). Clinical significance of serum mitochondrial DNA in lung cancer. Clin Biochem.

[33] Hosgood HD, Liu CS, Rothman N, Weinstein SJ, Bonner MR, Shen M (2010). Mitochondrial DNA copy number and lung cancer risk in a prospective cohort study. Carcinogenesis.

[34] Merritt RC, Manor U, Salles FT, Grati M, Dose AC, Unrath WC (2012). Myosin IIIB uses an actin-binding motif in its espin-1 cargo to reach the tips of actin protrusions. Current biology..

[35] Hakimi MA, Bochar DA, Schmiesing JA, Dong Y, Barak OG, Speicher DW (2002). A chromatin remodelling complex that loads cohesin onto human chromosomes. Nature.

[36] Hickey AJ, Chai CC, Choong SY, de Freitas CS, Skea GL, Phillips AR (2009). Impaired ATP turnover and ADP supply depress cardiac mitochondrial respiration and elevate superoxide in nonfailing spontaneously hypertensive rat hearts. Am J Physiol Cell Physiol.

[37] Comuzzie AG, Cole SA, Laston SL, Voruganti VS, Haack K, Gibbs RA (2012). Novel genetic loci identified for the pathophysiology of childhood obesity in the Hispanic population. PLoS One.

[38] Kuo HC, Li SC, Guo MM, Huang YH, Yu HR, Huang FC (2016). Genome-wide association study identifies novel susceptibility genes associated with coronary artery aneurysm formation in Kawasaki disease. PLoS One.

[39] Jin Q, Mao X, Li B, Guan S, Yao F, Jin F (2015). Overexpression of SMARCA5 correlates with cell proliferation and migration in breast cancer. Tumour Biol.

[40] Silva PN, Gigek CO, Leal MF, Bertolucci PH, de Labio RW, Payao SL (2008). Promoter methylation analysis of SIRT3, SMARCA5, HTERT and CDH1 genes in aging and Alzheimer's disease. J Alzheimers Dis.

[41] Stopka T, Zakova D, Fuchs O, Kubrova O, Blafkova J, Jelinek J (2000). Chromatin remodeling gene SMARCA5 is dysregulated in primitive hematopoietic cells of acute leukemia. Leukemia.

[42] Gong J, Zhu M, Chu M, Sun C, Chen W, Jin G (2014). Genetic variants in SMARC genes are associated with DNA damage levels in Chinese population. Toxicol Lett.

[43] Lopez S, Buil A, Souto JC, Casademont J, Blangero J, Martinez-Perez A (2012). Sex-specific regulation of mitochondrial DNA levels: genome-wide linkage analysis to identify quantitative trait loci. PLoS One.

[44] Workalemahu T, Enquobahrie DA, Tadesse MG, Hevner K, Gelaye B, Sanchez SE (2017). Genetic variations related to maternal whole blood mitochondrial DNA copy number: a genome-wide and candidate gene study. J Matern Fetal Neonatal Med.

[45] Han JW, Zheng HF, Cui Y, Sun LD, Ye DQ, Hu Z (2009). Genome-wide association study in a Chinese Han population identifies nine new susceptibility loci for systemic lupus erythematosus. Nat Genet.

[46] Chen X, Wu Q, Depeille P, Chen P, Thornton S, Kalirai H (2017). RasGRP3 mediates MAPK pathway activation in GNAQ mutant Uveal melanoma. Cancer Cell.

[47] Xu W, Fukuyama T, Ney PA, Wang D, Rehg J, Boyd K (2006). Global transcriptional coactivators CREB-binding protein and p300 are highly essential collectively but not individually in peripheral B cells. Blood.

[48] Campbell JD, Alexandrov A, Kim J, Wala J, Berger AH, Pedamallu CS (2016). Distinct patterns of somatic genome alterations in lung adenocarcinomas and squamous cell carcinomas. Nat Genet.

[49] Fergelot P, Van Belzen M, Van Gils J, Afenjar A, Armour CM, Arveiler B (2016). Phenotype and genotype in 52 patients with Rubinstein-Taybi syndrome caused by EP300 mutations. Am J Med Genet A.

[50] Morris AP, Voight BF, Teslovich TM, Ferreira T, Segre AV, Steinthorsdottir V (2012). Large-scale association analysis provides insights into the genetic architecture and pathophysiology of type 2 diabetes. Nat Genet.

