# Supplementary material for: Genetic variants in nuclear DNA along with environmental factors modify mitochondrial DNA copy number: a population-based exome-wide association study
Source: BMC Genomics. 2018 Oct 16;19:752. doi: 10.1186/s12864-018-5142-7 (PMC6192277; doi:10.1186/s12864-018-5142-7)
Supplement: Supplementary file 1 — Figure S1. The effects of smoking and PM2.5 exposure level on mtDNA copy number. The first column indicated that smokers have higher mtDNA copy number than non-smokers. The 2–5 columns showed that the median mtDNA copy number in subjects with low and high PM2.5 exposure in each city and combined analysis. (DOCX 331 kb) [file 12864_2018_5142_MOESM1_ESM.docx]

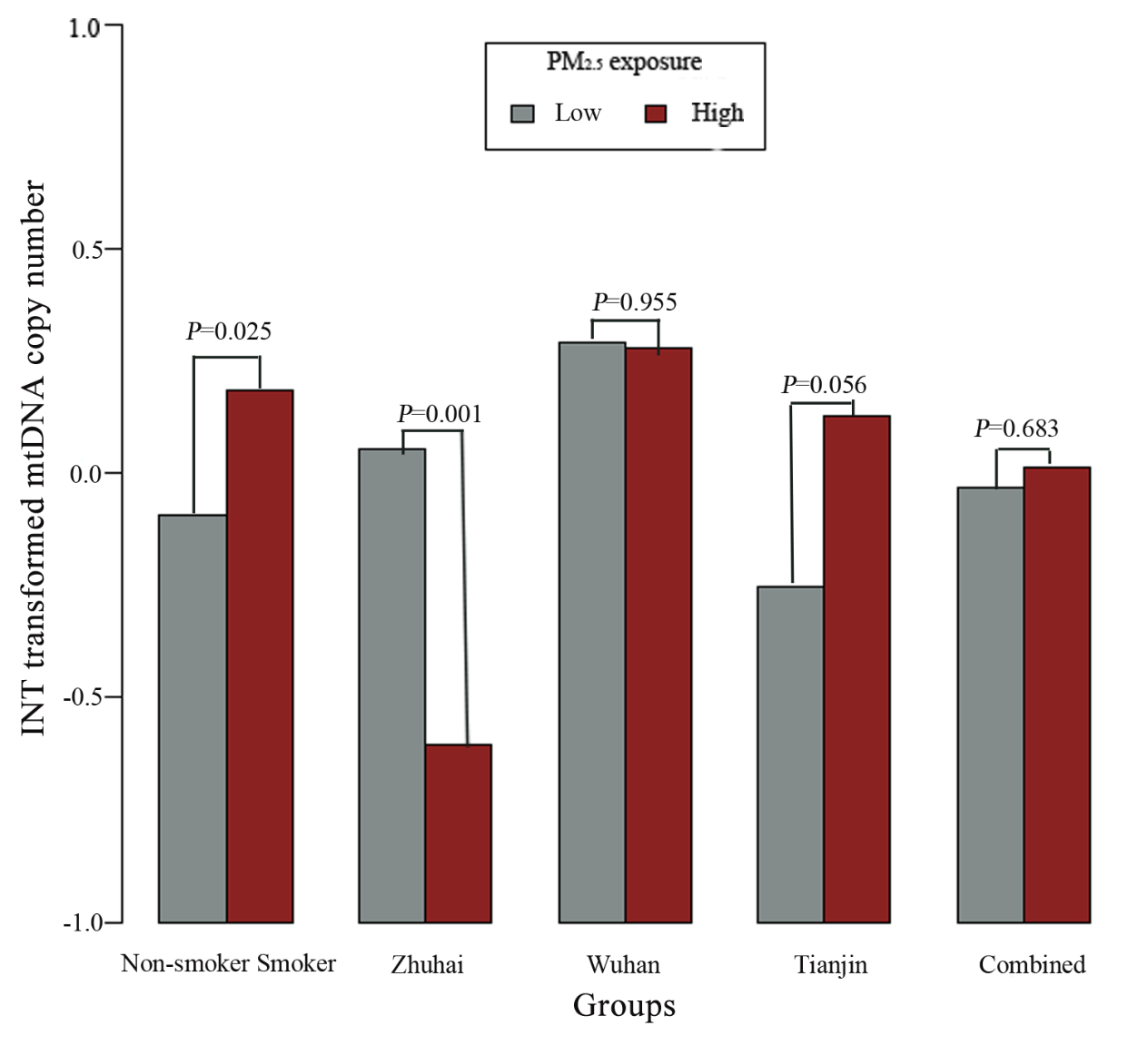


**Figure S1**. The effects of smoking and PM_2.5_ exposure level on mtDNA copy number. The first column indicated that smokers have higher mtDNA copy number than non-smokers. The 2-5 columns showed that the median mtDNA copy number in subjects with low and high PM_2.5_ exposure in each city and combined analysis.
