# Supplementary material for: Genetic variants in nuclear DNA along with environmental factors modify mitochondrial DNA copy number: a population-based exome-wide association study
Source: BMC Genomics. 2018 Oct 16;19:752. doi: 10.1186/s12864-018-5142-7 (PMC6192277; doi:10.1186/s12864-018-5142-7)
Supplement: Supplementary file 2 — Table S1. Association between PM2.5, smoking and mtDNA copy number. Table S2. Functional annotations for our identified 7 significant SNPs. (DOCX 21 kb) [file 12864_2018_5142_MOESM2_ESM.docx]

**Table S1.** Association between PM_2.5_ exposure level, smoking and mtDNA copy number.

| **Variables** | **Zhuhai** | |  | **Wuhan** | |  | **Tianjin** | |  | **Meta** | |
| --- | --- | --- | --- | --- | --- | --- | --- | --- | --- | --- | --- |
|  | **β** ^a^ | ***P*** |  | **β** ^a^ | ***P*** |  | **β** ^a^ | ***P*** |  | **β** ^a^ | ***P*** |
| PM_2.5_ | -3.69×10^-3^ | 0.024 |  | 6.98×10^-4^ | 0.692 |  | -3.50×10^-4^ | 0.535 |  | -6.00×10^-4^ | 0.241 |
| Smoking | 0.417 | 0.247 |  | 0.521 | 0.132 |  | 0.424 | 0.100 |  | 0.448 | **0.012** |
| Pack-years | 0.016 | 0.095 |  | -1.35×10^-3^ | 0.860 |  | 0.010 | 0.106 |  | 0.008 | 0.072 |

^a^ Linear regression model was adopted to perform the association analysis; Age, gender, smoking pack-years and PM_2.5_ exposure level were adjusted when appropriate.

**Table S2.** Functional annotations for our identified 7 SNPs.

| **Chr** | **SNP** | **Gene** | **RegulomeDB** ^a^ | **CADD** ^b^ | **Motifs changed** ^c^ | **eQTL** ^d^ |
| --- | --- | --- | --- | --- | --- | --- |
| 5 | rs37576 | *PDE4D* | 7 | 7.44 | EBF, MOVO-B | *PDE4D* |
| 13 | rs7326068 | *IFT88* | 5 | 0.75 | Evi-1 | *SLC35E1P1*, *XPO4*, *GRK6P1*, *IFT88*, *CRYL1* |
| 8 | rs7000642 | *IFITM8P* | 7 | 2.18 | ATF3 | CTD-3046C4.1 |
| 13 | rs9507174 | *MIPEP* | 7 | 8.46 | Cdx, Irf_disc3, Pax-4_5, Pdx1_1, Pou2f2_known11, Pou3f2_2, STAT_disc3, Sox_16 | *MIPEP*, *SACS-AS1*, *C1QTNF9B-AS1*, *C1QTNF9* |
| 1 | rs2780886 | *JAK1* | 6 | 1.03 | MEF-2 | *JAK1*, *RP11-182I10.3* |
| 2 | rs33962844 | *MYO3B* | 5 | 13.98 |  | *MYO3B* |
| 4 | rs6857360 | *SMARCA5* | 7 | 3.51 | Ets, SP2 | *SMARCA5, GUSBP5, RP11-673E1.1* |

^a^ The RegulomeDB score based on RegulomeDB (<http://regulome.stanford.edu/>) website; 5 represented in TF binding or DNase peak; 6 represented the others; 7 was no data;

^b^ The CADD score using http://cadd.gs.washington.edu/home website, larger values indicated more harmful to the human genomes;

^c^ The changed motifs according to HaploReg v4.1 website (http://archive.broadinstitute.org/mammals/haploreg/haploreg.php);

^d^ Based on GTEx V7 database in multiple tissues (http://www.gtexportal.org/home/);
