# Supplementary material for: Genetic variants in nuclear DNA along with environmental factors modify mitochondrial DNA copy number: a population-based exome-wide association study
Source: BMC Genomics. 2018 Oct 16;19:752. doi: 10.1186/s12864-018-5142-7 (PMC6192277; doi:10.1186/s12864-018-5142-7)
Supplement: Supplementary file 3 — Figure S2. (a) Association between rs9507174 genotypes and mtDNA copy number; (b): Association between rs9507174 genotypes and the expression of MIPEP gene according GTEx database; (c): Association between rs9507174 genotypes and lung cancer risk using our previous data. The frequency of “A” allele of rs9507174 was higher in lung cancer patients; (d): Association between mtDNA copy number and lung cancer risk. This figure was made based on the result from Hosgood et al. ‘s paper. The horizontal axis was the quartile of mtDNA copy number and the vertical axis represents the Odds Ratio for lung cancer. (DOCX 496 kb) [file 12864_2018_5142_MOESM3_ESM.docx]

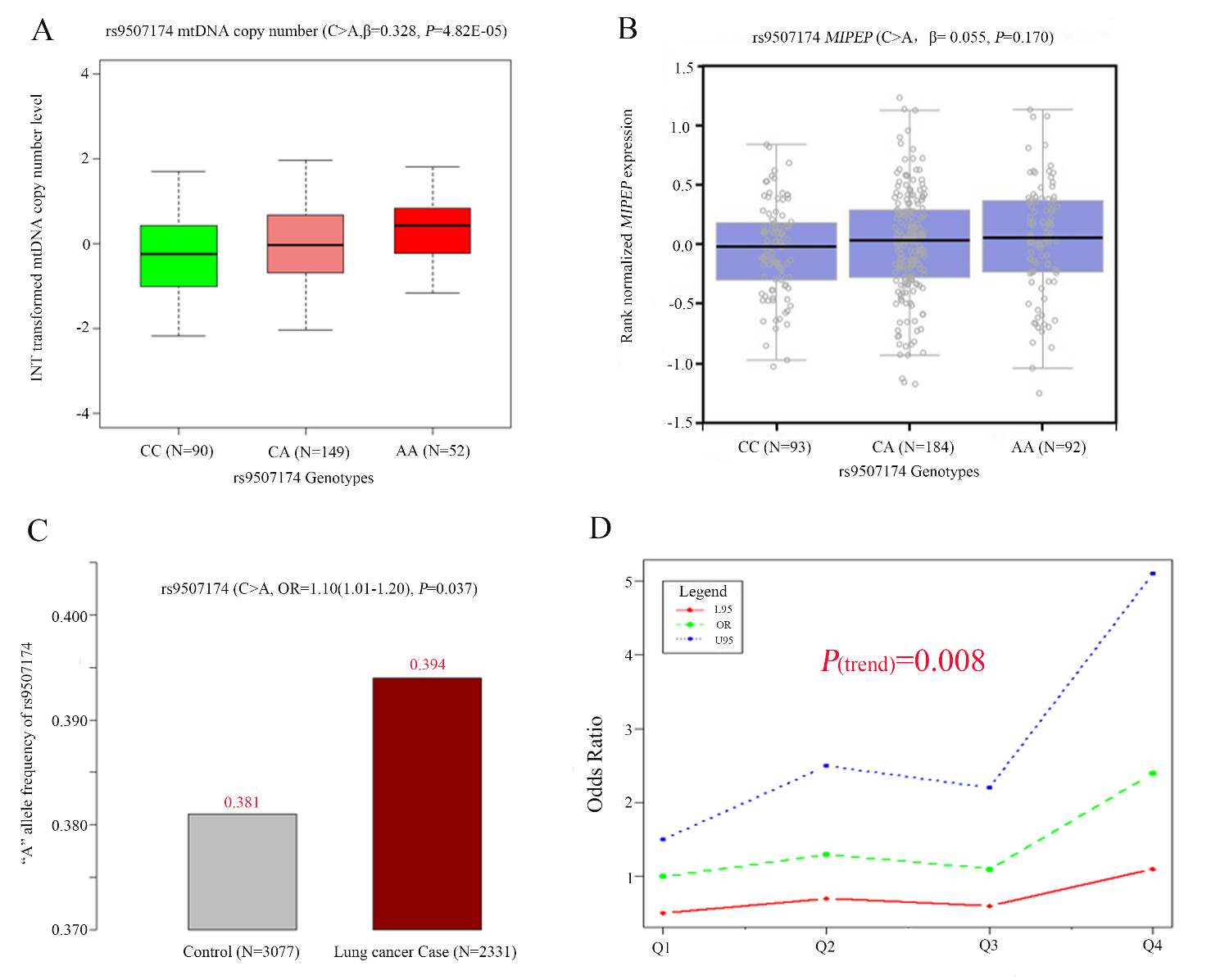


**Figure S2**. (**A**) Association between rs9507174 genotypes and mtDNA copy number; (**B**): Association between rs9507174 genotypes and the expression of *MIPEP* gene based on GTEx database; (**C**): Association between rs9507174 genotypes and lung cancer risk using our previous data. The frequency of “A” allele of rs9507174 was higher in lung cancer patients; (**D**): Association between mtDNA copy number and lung cancer risk. The (**D**) figure was made based on the result from Hosgood *et al* ‘s paper. The horizontal axis was the quartile of mtDNA copy number and the vertical axis represents the Odds Ratio for lung cancer.
